# Longitudinal associations of parent-child communication, dating behaviors, decision-making processes, and sex initiation among United States Latina/o adolescents

**DOI:** 10.3389/fpsyg.2022.897311

**Published:** 2022-08-12

**Authors:** Patricia Cabral, Jan L. Wallander, Marc N. Elliott, Mark A. Schuster

**Affiliations:** ^1^Department of Psychology, Occidental College, Los Angeles, CA, United States; ^2^Psychological Sciences and Health Sciences Research Center, University of California, Merced, Merced, CA, United States; ^3^RAND Corporation, Santa Monica, CA, United States; ^4^Kaiser Permanente Bernard J. Tyson School of Medicine, Pasadena, CA, United States

**Keywords:** parent-child sex communication, dating behaviors, sex initiation, Latino, decision making, adolescent

## Abstract

**Objective:**

This study examined differences and identified developmental predictors of oral, vaginal, and anal intercourse initiation across generational status among Latina/o adolescents of both genders. More specifically, we compare generational status and gender differences in the longitudinal predictions from parent-child sex communication and dating behaviors to sex initiation 5 years later, and how these associations may be mediated by perceived peer norms, intentions, and attitudes regarding sex among Latina/o adolescents.

**Methods:**

Using prospective longitudinal data from the *Healthy Passages™* project collected in Houston and Los Angeles, Latina/o girls (*n* = 879) and boys (*n* = 885) who were identified as 1st- (18%), 2nd- (58%), or 3rd (24%)-immigrant generational status reported on their dating behaviors and parent-child communication about sex at 5th grade (*M* age = 11.12 years), their perceived peer norms and attitudes regarding sex at 7th grade (*M* age = 13.11 years), and if they had initiated oral, vaginal, or anal sexual intercourse by 10th grade (*M* age = 16.06 years).

**Results:**

Third-generation Latina girls were more likely than 1st- and 2nd-generation Latinas to have initiated sexual intercourse by 10th grade. More advanced dating behaviors in 5th grade had a positive association with sex initiation for all generational status groups among Latino boys, but only among 1st-generation Latina girls. Moreover, mediating decision-making processes of peer norms and attitudes differed for each group.

**Conclusion:**

Pre-adolescent dating behaviors are associated with long-term differences in adolescents’ sexual behaviors, which may point to targets for prevention efforts. Acculturation differences may contribute to different ways in which adolescents decide to engage in sexual intercourse based on the previous dating experiences.

## Introduction

Early sexual intercourse initiation, defined in the United States (US) as sexual debut before age 16 (Madkour et al., 2010; Epstein et al., 2018), is associated with engagement in other sexual risk behaviors (e.g., inconsistent condom use and multiple sexual partners; O’Donnell et al., 2001; Akumiah et al., 2020). Latina/o youth are twice as likely as their non-Latina/o White peers to report engaging in sexual intercourse before age 13 (Centers for Disease Control and Prevention (CDC), 2014; Moilanen et al., 2018). Moreover, their sexually transmitted infection (STI) prevalence is more than twice that of non-Latino Whites (Centers for Disease Control and Prevention (CDC), 2019). Therefore, delaying sex initiation among US Latina/o youth may have significant public health implications by reducing sexual risk behaviors.

To develop better prevention strategies, researchers have endeavored to identify psychosocial influences on delaying sex initiation (Scott et al., 2014; Rotz et al., 2020). However, these influences are often confounded by acculturation processes in which cultural, behavioral, and psychological changes take place as a result of contact between culturally dissimilar people (Berry, 2006; Schwartz et al., 2010). Specifically, Latina adolescents more acculturated to the mainstream US culture are more likely to initiate sexual intercourse before the end of high school than those who are less acculturated, in this way appearing more similar to White adolescents (Cabral et al., 2017). Yet, it remains unclear how these differences occur and may be affected by parents and peers and individual decision-making processes among Latino youth. To that end, we examine here how parent communication about sex, early dating behaviors, and decision-making processes may influence sexual intercourse initiation among Latino/a adolescents of both genders and at different acculturation levels.

### Parent-child communication about sex

Parents are an important source of sexual health information for youth. However, most Latino parents in the US do not talk comprehensively about sex or dating with their teens and are often unwilling to acknowledge the possibility of their child having sex (Guzman et al., 2013; Martinez and Orpinas, 2016). Latino parents often report difficulty communicating about sex, and tend to talk about sex with their daughters more than with their sons mainly due to pregnancy concerns (Raffaelli and Ontai, 2001; Hoff et al., 2003; Raffaelli and Green, 2003; Jerman and Constantine, 2010; Ashcraft and Murray, 2017). Yet, adolescents, who are aware of this dynamic, want their parents to deliver the same message to sons and daughters (Guilamo-Ramos et al., 2006; Vexler, 2007; Guilamo-Ramos and Bouris, 2008). Another challenge is that parents whose expectations and values differ from those of the majority culture, as is the case for many Latino families in the US, are less likely and more reluctant to talk to their children about sex than parents of other ethnic groups (Guilamo-Ramos et al., 2006; Kenny and Wurtele, 2013; Lantos et al., 2019) due to a perceived lack of expertise in the topic (Martinez and Orpinas, 2016). Nevertheless, when parents and youth have good communication, youth initiate intercourse later (Beckett et al., 2010; Coakley et al., 2017). Therefore, the association between parent-child communication about sex and sexual intercourse initiation should be examined among Latina/o adolescents.

### Dating behaviors and sex initiation

In American society, dating and romantic relationships are considered a hallmark of adolescents’ healthy development (Collins, 2003; Furman and Rose, 2015; Chang and Rosenthal, 2018). However, when adolescents begin dating exclusively (going steady) at a younger age, they are more likely to engage in sexual activity at an earlier age (Collins et al., 2009; Gordon and DeLamater, 2015; Lee et al., 2018). In fact, romantic relationships are the context in which most US adolescents’ sexual behavior occurs (Muise et al., 2018). Among US Latino families, parents are particularly cautious regarding their children’s dating practices. They often prohibit or impose stringent rules regarding dating as strategies to protect adolescents, especially daughters from premature sexual involvement (Raffaelli and Ontai, 2001; Raffaelli, 2005; Haglund et al., 2019).

Many Latino parents are apprehensive about US-style dating, which has evolved into more brief sexual encounters and a de-emphasis on traditional, committed relationships (Garcia et al., 2012; MIT International Students Office, 2021). Likewise, Latino parents are often uneasy about the majority culture in the US valuing independence among adolescents rather than adolescents being influenced more by family (Garcia et al., 2012; MIT International Students Office, 2021). For these reasons, early dating experiences among Latina/o youth often occur without parental knowledge (Raffaelli and Ontai, 2001; Raffaelli, 2005). Despite these differences, the link between early dating behaviors and sexual intercourse initiation specifically among Latina/o youth does not appear to have been examined.

### Dating behaviors and parent-child communication about sex

Studies suggest that parents should begin talking to their children about sex before they start dating (Guzmán et al., 2003; O’Donnell et al., 2006; Pariera and Brody, 2018). Many US parents report discussing dating and relationships with their children prior to discussions about sex (Grossman et al., 2018). In fact, dating behaviors among their children may be a motivating factor for parents to begin discussing sex with their teens. However, most Latino parents underestimate their children’s engagement in sexual activity (Guzmán et al., 2006; O’Donnell et al., 2008; Scull et al., 2022) and may, therefore, underestimate the onset of and engagement in dating behaviors. Thus, it remains unclear if parent-child discussions about sex is associated with dating behaviors among Latina/o youth.

### Cognitive decision-making processes in sex initiation

Social influences, such as from parents and peers, must be mediated by cognitive decision-making processes regarding sexual behaviors. Specifically, according to the Theory of Planned Behavior, intentions, attitudes, and perceptions of peer norms (Ajzen, 1991) are major cognitive determinants of adolescent decisions regarding sexual behaviors (Halpern-Felsher et al., 2005; Buhi and Goodson, 2007; Kalolo and Kibusi, 2015; Lin et al., 2021). For example, Spanish language-dominant US Latina/o youth who reported intentions to have sexual intercourse in the next 3 months were more likely to have had sexual intercourse in the following 3 months (Villarruel et al., 2004). Moreover, intentions are determined by attitudes about behaviors and perceptions of peer norms (Ajzen, 1991). Indeed, adolescents are more likely to initiate sexual intercourse if they have permissive or positive attitudes toward sex (O’Donnell et al., 2003; Cuffee et al., 2007; Cox et al., 2015; Coyne et al., 2019). Also, when US adolescents perceive their peers to be engaging in sexual activity, they tend to have higher intentions to initiate sexual intercourse and are more likely to initiate sexual intercourse (Flores et al., 2002; Cox et al., 2015). However, it is important to examine these associations specifically among Latina/o youth in the US, as sexual attitudes and intentions may differ compared to non-Latino youth or their more acculturated peers (Killoren et al., 2011a). For example, Latina/o adolescents are more resistant to peer pressure to engage in antisocial behaviors than their US-born counterparts (Bamaca and Umaña-Taylor, 2006; Sanchez et al., 2020). Thus, it is possible that peers play a stronger role in adolescents’ sexual decision-making for Latina/o youths born in the US than for those born outside the US.

Given the centrality of the family in the Latino culture (Kulish et al., 2019), it is likely that parent-child communication about sex plays a role in shaping Latina/o adolescents’ sexual decision-making processes (Malcolm et al., 2013). For example, parent-child communication about sex is associated with youth’s sexual risk attitudes (Lederman et al., 2004; Hurst et al., 2022). Also, engagement in dating behaviors may influence decision-making processes regarding sexual intercourse initiation. In fact, among Latina/o adolescents, being in a romantic relationship is associated with higher intentions to have sex (Guilamo-Ramos et al., 2009). Yet, the influence of early dating behaviors on subsequent attitudes about sex and sex initiation among Latino youth remains unclear.

**FIGURE 1:**
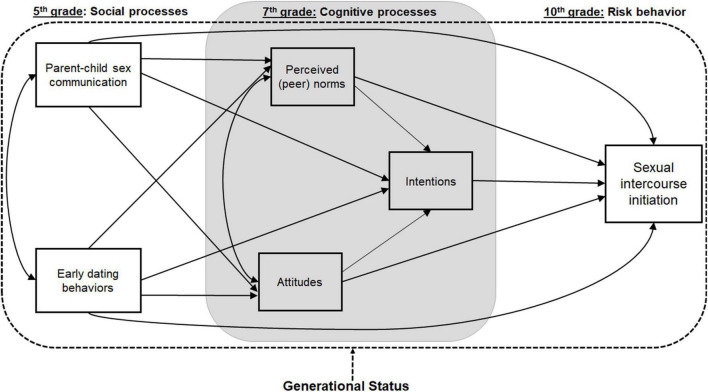
Conceptual hypothesized model.

To support healthy sexual outcomes among Latina/o adolescents, we must understand the role of both social influences and decision-making processes for sexual behaviors. Specifically, we propose a conceptual model (see Figure 1) of how parent-child discussions about sex and adolescents’ pre-adolescent dating behaviors may influence sexual intercourse initiation through the adolescents’ decision-making processes, including attitudes and peer norms regarding sex. However, these associations may be further compounded by Latino adolescents’ acculturation to the US, which has been associated with different rates of sexual risk behaviors (Afable-Munsuz and Brindis, 2006; Lee and Hahm, 2010; Cabral et al., 2017; Ertl et al., 2018).

### Immigrant paradox in sex initiation

Acculturation typically follows migration, which typically brings about changes in attitudes, beliefs, and behaviors that unfold across time and generations. Acculturation is typically associated with when an individual or a family arrived in the US and occurs as a process of change in cultural patterns that result from continuous firsthand contact between people from different cultures (Redfield et al., 1936; Berry, 1997). In fact, previous studies reveal a pattern of associations between cultural orientations and acculturation indices, including generational status (Umaña-Taylor et al., 2009; Miller, 2010; Testa et al., 2019). The acculturation process of immigrant families may contribute to challenges experienced by Latino families, including language barriers, depleted social resources, and experiences of discrimination. In fact, immigration and associated acculturation processes play an important role in the experience of many Latino families. Paradoxically, recently immigrated individuals tend to have an initial health advantage, despite having a predominantly lower socioeconomic status which is usually associated with poorer health outcomes (Franzini et al., 2001). Then, as children in immigrant families acculturate to the US, their health outcomes appear to become less favorable, a phenomenon termed the *immigrant paradox* (Coll and Marks, 2012). This pattern is especially salient for health risk behaviors among less acculturated generations, immigration generational status being a common proxy variable for acculturation. In fact, 1st-generation Latina/os, who themselves migrated to the US, have been found less likely to engage in sexual risk behaviors, such as later age of first intercourse (Cabral et al., 2017), higher condom use, and lower number of sexual partners (Guarini et al., 2011), than Latina/os who are born in the US.

Social and cognitive processes may illuminate these paradoxical findings across the generational status. Specifically, parental and peer factors have both been found to mediate and moderate associations between Latina/o youth acculturation in the US and sexual behaviors (e.g., Cabral et al., 2017). For example, among the 1st- and 2nd-generation Latino youth, lack of maternal communication about sex is a key predictor of risky sexual behaviors (Trejos-Castillo and Vazsonyi, 2009; Estrada-Martínez et al., 2021). Moreover, decision-making processes appear to differ across acculturation levels, in which Latina/o youth with higher acculturation tend to express more positive attitudes toward casual sex (Ahrold and Meston, 2010; Blanc, 2021). This may be further compounded by dating behaviors. For example, among higher acculturated adolescents, a lower likelihood of being in a romantic relationship is associated with lower intentions to have sex (Guilamo-Ramos et al., 2009). Yet, these associations have not been examined together in a single developmental model for sexual intercourse initiation among Latina/o adolescents across different generational statuses, especially considering the spectrum of oral, vaginal, and anal intercourse.

### Current study

This study examined differences in and developmental correlates of oral, vaginal, and anal intercourse initiation across generational status among US Latina/o adolescents using a longitudinal design. Specifically, we hypothesized that (**H1**) less acculturated Latina/os, as indicated by generational status, will report a lower prevalence of sexual intercourse initiation by 10th grade. Moreover, we examined hypotheses about sexual intercourse initiation among Latina/o youth based on a conceptual model depicted in Figure 1, including (**H2**) early parent-child sex communication and pre-adolescent dating behaviors will be, respectively, negatively and positively associated with sexual intercourse initiation by 10th grade; and (**H3**) intentions to initiate sex, perceived peer norms, and attitudes about sex will mediate these relationships. Due to differences across acculturation for sexual intercourse initiation, we explored, in the absence of a strong basis for forming hypotheses, how these relationships (see H2 and H3) varied by generational status.

## Materials and methods

Data came from *Healthy Passages™*, a multisite longitudinal study of health behaviors and outcomes, and associated risk and protective factors (see; Windle et al., 2004; Schuster et al., 2012), prospectively following a cohort across 5th (assessed 2004–2006), 7th, and 10th (2009–2011) grades. In this manner, the *Healthy Passages™* data collection was designed to begin prior to the initiation of most risk behaviors in the vast majority of cases. Specific to sex initiation, less than 4% of US adolescents initiate sex before the age of 13 (2.1% females, 3.9% males) according to the National Youth Risk Behavior Survey (YRBS), a school-based survey measuring adolescent health risk behaviors and experiences that is conducted every other year among a nationally representative sample of US public and private high school students. Centers for Disease Control and Prevention (CDC), as the funding agency, invited proposals from an existing network of sites distributed nationally to become a research site. Of these, research sites in Birmingham, Alabama; Houston, Texas; and Los Angeles, California were selected and deemed to be best qualified based on a range of factors, one of which was that they collectively could recruit around 6,000 youth participants evenly distributed among the three largest racial/ethnic groups in the US (i.e., Black, Latino/a, and White). All instruments were tested in a pilot study including over 600 diverse participants in the relevant age range and subjected to analysis to support their use in the Healthy Passages*™* study.

### Sampling procedures and participants

Sampling for the Healthy Passages study included 5th graders in regular public school classrooms in three sites (Birmingham, Alabama; Houston, Texas; and Los Angeles, California). Child participants were selected using a two-stage probability sampling procedure. Public schools within the study sites were randomly selected with probabilities proportionate to a weighted measure of the scarcity of a school’s students relative to race/ethnicity targets to ensure adequate sample sizes of Black, Latina/o, and White students, the three largest racial/ethnic groups in the US. All 5th-grade students in regular classrooms within selected schools during two consecutive school years (2004–2005 and 2005–2006) were invited to participate (see Schuster et al., 2012). There were no exclusion criteria. Among families who provided permission to be contacted and completed interviews in 5th grade (*N* = 5,147; 2,607 girls; 1,813 Latina/o), 4,773 (93%) and 4,521 (89%) completed the 7th and 10th grade assessments, respectively. Retention was 89% by 10th grade.

Because child participants were not presented with dating behavior questions at Wave 1 and the very low number of Latino/a youth at the Birmingham site (*n* = 26), the data analytic sample (*n* = 1,764, 49.8% girls) consisted of those from the Los Angeles and Houston sites who completed all three waves, and could be classified as first- (18%), second- (58%), or third- (24%) generation subjects (*n* = 23 could not be classified). The 49 Latina/o participants who were thus excluded did not differ from the analysis sample in any demographics. Final sample mean age = 11.13 years (*SD* = 0.59) at 5th grade, 13.11 years (0.61) at 7th grade, and 15.66 years (0.65) at 10th grade. Selected sample characteristics are presented in Table 1 (see Schuster et al., 2012, for more details).

**TABLE 1:** Descriptive characteristics for the overall sample (*N* = 1,764) and by gender and generational status.

	Overall	Latino boys			Latina girls		
		1st Gen.	2nd Gen.	3rd Gen.	1st Gen.	2nd Gen.	3rd Gen.
	*M (SD)*	*M (SD)*	*M (SD)*	*M (SD)*	*M (SD)*	*M (SD)*	*M (SD)*
**Parent-child sex communication (5th gr)^c^**							
Mom: How babies are made	1.91 (0.74)	1.63 (0.72)^a1^	1.57 (0.66)^a1^	1.57 (0.64)^a1^	1.98 (0.74)^a2^	1.92 (0.75)^a,b2^	1.84 (0.69)^b2^
Mom: What is sex	1.67 (0.76)	1.46 (0.71)^a1^	1.40 (0.64)^a1^	1.44 (0.60)^a1^	1.77 (0.80)^a2^	1.65 (0.76)^b2^	1.64 (0.74)^a2^
Mom: Wait to have sex	2.02 (0.89)	1.85 (0.86)^a1^	1.78 (0.86)^a1^	1.70 (0.81)^a1^	2.11 (0.86)^a2^	2.03 (0.90)^a2^	1.90 (0.88)^b2^
Dad: How babies are made	1.35 (0.60)	1.57 (0.74)^a1^	1.45 (0.66)^b1^	1.40 (0.58)^b1^	1.49 (0.68)^a1^	1.33 (0.58)^b2^	1.30 (0.56)^b1^
Dad: What is sex	1.30 (0.58)	1.44 (0.68)^a1^	1.35 (0.62)^a,b1^	1.30 (0.55)^b1^	1.37 (0.61)^a1^	1.30 (0.60)^a,b1^	1.22 (0.50)^b1^
Dad: Wait to have sex	1.62 (0.82)	1.78 (0.86)^a1^	1.66 (0.82)^a,b1^	1.58 (0.77)^b1^	1.76 (0.82)^a1^	1.63 (0.83)^b1^	1.48 (0.76)^c1^
**Attitudes (7th gr)^d^**							
Sex with casual friend	1.80 (0.91)	2.19 (1.03)^a1^	2.07 (1.0)^a1^	2.07 (0.94)^a1^	1.47 (0.74)^a2^	1.52 (0.73)^a2^	1.55 (0.73)^a2^
Sex with boy-friend/girl-friend	1.89 (0.95)	2.24 (1.07)^a1^	2.08 (0.98)^a,b1^	2.04 (0.93)^b1^	1.55 (0.83)^a2^	1.71 (0.87)^b2^	1.70 (0.86)^a2^
Sex acceptable with condom	2.10 (1.03)	2.37 (1.08)^a1^	2.31 (1.01)^a1^	2.34 (1.02)^a1^	1.81 (1.0)^a,b2^	1.96 (1.0)^b2^	1.74 (0.89)^a2^
Sex acceptable in love	1.97 (0.97)	2.23 (1.07)^a1^	2.18 (1.01)^a1^	2.22 (0.95)^a1^	1.72 (0.89)^a,b2^	1.80 (0.90)^b2^	1.63 (0.82)^a2^
Sex acceptable if older than 18	2.43 (0.95)	2.59 (0.98)^a1^	2.59 (0.92)^a1^	2.65 (0.94)^a1^	2.17 (0.87)^a2^	2.28 (0.93)^a2^	2.26 (1.0)^a2^
Intentions (7th gr)^e^	1.55 (0.96)	1.90 (0.96)^a1^	1.79 (0.93)^a1^	1.72 (1.02)^a1^	1.04 (0.87)^a2^	1.26 (0.86)^b2^	1.42 (0.88)^b2^
	%	%	%	%	%	%	%
**Dating behaviors (5th gr)**							
Held hands	20.2	27.6^a1^	27.0^a1^	28.6^a1^	11.0^a2^	12.3^a2^	15.9^a2^
Alone with boy-friend/girl-friend	13.2	16.7^a1^	17.9^a1^	22.6^a1^	6.9^a2^	5.9^a2^	13.9^b2^
Kissed on mouth	11.2	14.8^a1^	12.5^a1^	18.4^a1^	7.8^a2^	8.0^a2^	9.4^a2^
Said “I love you”	17.4	22.7^a1^	22.3^a1^	28.2^a1^	9.7^a2^	10.8^a2^	13.9^a2^
Hands under clothes	1.5	0.0^a1^	2.1^a1^	2.1^a1^	2.3^a2^	0.8^a2^	2.4^a1^
Peer norms (7th gr)^f^	23.7	29.6^a,b1^	23.6^a1^	31.6^b1^	17.9^a2^	20.5^a1^	26.0^a1^

Gen., generational status; k, thousand; yr, year; bf/gf, boyfriend/girlfriend. Superscript letters across rows represent significant differences across generational status within gender.

Superscript numbers across rows represent significant differences between gender within generational status. Prevalence reported represent weighted percentages.

^c^ Range = 1–3.

^d^ Range = 1–4.

^e^ Range = 0–3.

^f^ Peer norms of vaginal intercourse (VI) initiation indicate the percentage who reported at least one friend to have initiated sex at 7th grade.

### Data collection procedure

Institutional review boards at each study site and the CDC approved the study. At each of the three assessment waves, two trained interviewers completed the full *Healthy Passages™* assessment protocol with an adolescent and their primary parent or caregiver (mother, 88%; father, 6%; and other, 6%) at their home or a research facility. The parent provided signed informed consent, and the adolescent signed assent at each assessment. The adolescent and parent were then separated and assessed individually in private spaces. Interviews were conducted using two formats (see Windle et al., 2004 for details). The larger portion of items was administered using a computer-assisted personal interview (CAPI) procedure administered by an interviewer. Sensitive data, including information on sexual behaviors used in this study, were instead collected by computer-assisted audio self-interview (CASI) method, where the participant responds to items completely on his/her own without involvement by the interviewer. A Spanish version could be chosen by participants at each assessment, except for adolescents in 10th grade (applied partly or fully at 5th grade: 8% of adolescents, 23% of parents; 7th grade: 4% of adolescents, 30% of parents; and 10th grade: 30% of parents). The Spanish version was developed using standard back-translation and verification by bilingual staff experienced with this procedure.

### Measures

#### Sexual intercourse initiation

Adolescents indicated in the 10th-grade assessment whether they had ever performed or received oral sex, vaginal intercourse, or anal intercourse, in separate questions, and those who responded yes to any were coded as having initiated sexual intercourse by 10th grade (Yes = 1, No = 0). Reported sexual intercourse included same-sex experiences.

Parent-Child Communication about Sex (items adapted from Dittus et al., 2004). Adolescents reported at 5th-grade assessment about their discussions with each parent with two items about reproduction (*How many times has your mother/father ever talked to you about how babies are made or where babies come from?*) and four items about sexual activity (e.g., *How many times has your mother/father ever told you that you should wait to have sex until you are married?*α = 0.83). Responses were made on a three-point scale (*1* = *Never talked about it*; *2* = *Talked about it once or twice*; *3* = *Talked about it lots of times*). Based on structural equation modeling (SEM) measurement model analysis (see details in Supplementary Appendix A), individual items were entered as observed variables in the analysis to indicate the latent factor of parent-child communication about sex.

Dating Behaviors (items adapted from Dittus et al., 2004). Adolescents indicated in 5th- grade assessment if they had ever: (a) held hands with a boyfriend/girlfriend, (b) told a boyfriend/girlfriend they love him/her, (c) kissed a boyfriend/girlfriend, (d) been left alone with a boyfriend/girlfriend, or (e) had their hands under a boyfriend/girlfriend’s clothes or vice versa (α = 0.82). Responses for each item were dichotomized into *no* (0) or *yes* (1). Based on SEM measurement model analysis (see details in Supplementary Appendix A), individual items were entered as observed variables in the analysis to indicate the latent factor of dating behaviors.

Sexual Decision-Making Processes (items adapted from Schuster et al., 2006) were measured in 7th grade. Intentions were measured as a single item based on the adolescent’s response to whether they intended to wait until the end of high school or marriage to have [vaginal] intercourse (*Do you intend to wait until the end of high school before having vaginal intercourse [again]?*). Responses were on a four-point scale (*0* = *Yes, definitely* to *3* = *No, definitely not*). Perceived peer norms were assessed with a single item (*How many of your friends have had oral or vaginal sex?*). Responses were dichotomized (*0* = *None*; *1* = *at least one friend*). Adolescents reported their attitudes about sexual intercourse indicating their agreement with five statements regarding sex (e.g., *It is ok for people your age to have vaginal intercourse with a casual friend*; *It is ok for people your age to have vaginal intercourse if they are in love*; α = 0.84), using a four-point Likert scale (*1* = *Strongly disagree* to *4* = *Strongly agree*). Based on SEM measurement model analysis (see details in Supplementary Appendix A), these five individual items were entered as observed variables in the analysis to indicate the latent factor of parent-child communication about sex.

#### Generational status

Parents were asked if they and the adolescent were born inside or outside the US. Using a common classification scheme (Coll and Marks, 2012), the adolescent was classified as (a) first generation if both were born outside the US; (b) second generation if the adolescent was born inside but the parent was born outside the US; and (c) third generation if both were born inside the US.

#### Covariates

We controlled for adolescents’ age at 5th grade, total household income (nine categories), and one- vs. two-parent household for extraneous influence on early dating behaviors and parent-child sex communication. Additionally, we controlled for adolescents’ age at 10th grade when sexual intercourse initiation was the outcome.

### Statistical analysis

All analyses were performed using sampling weights to account for the survey design, including the effects of design, non-response, and attrition over time; clustering of youth within schools in each area; and stratification by site (see Schuster et al., 2012). Consequently, the weighted results reported here adjust for differential attrition over time and represent the population in the sampling frame of the three defined communities. Missing responses to sex initiation in Wave 3 (<10%) were addressed using responses from previous waves. All analyses were conducted separately for girls and boys due to marked differences in sexual intercourse initiation between gender. Using *SPSS v.24*, chi-square analysis and *t*-tests were used to examine differences among observed variables across generational status, including in sexual initiation to address hypothesis 1. Applying a structural equations modeling (SEM) framework using *Mplus v.7*, a series of sequential tests were conducted to address hypotheses 2 and 3 and the final exploratory aim, in each case separately for girls and boys. SEM analysis can integrate both parametric and non-parametric variables in one analysis, while details about the analysis will be provided as results are reported; as an overview, first, we conducted a confirmatory factor analysis to assess the adequacy of the measurement model, followed by measurement invariance (or equivalency) tests. Measurement model testing and results are reported in Supplementary Appendix A.

Subsequently, we tested the overall structural equation model among the whole sample of Latina girls and Latino boys. Finally, to investigate whether generational status moderated the hypothesized relationships, a multiple-group SEM analysis was conducted (Kline, 2005; Byrne, 2012), testing whether path coefficients between latent factors of the hypothesized model (Figure 1) differed across generation status groups. Again, these were conducted separately for girls and boys. SEM analyses controlled for household income, household parental composition, and age at 5th grade on the exogenous variable of parent-child sex communication and dating behaviors, as well as age at 10th grade on the outcome of sexual initiation. Standard criteria were applied (Hu and Bentler, 1998) when examining three goodness-of-fit indices [Comparative Fit Index (CFI), Tucker Lewis Index (TLI), and Root Mean Square Error of Approximation (RMSEA)] to determine how the model reproduced the observed data.

## Results

Descriptive statistics across groups for variables of interest are presented in Table 1. The prevalence of sex initiation across demographic and categorical predictive variables is shown in Table 2. See Supplementary Appendix B for intercorrelations.

**TABLE 2:** Prevalence of sexual intercourse (oral, vaginal, and anal) initiation by demographic and categorical predictors.

		Overall sample (%)	Latino boys (%)	Latina girls (%)
Overall sexual initiation^a^		33.1	39.3	27.0
	Oral sex initiation	26.8	34.5	19.2
	Vaginal sex initiation	26.4	31.3	21.5
	Anal sex initiation	9.7	13.3	6.1
Generational status		**χ^2^ (2) = 8.10 (*p* = 0.02)**	χ^2^ (2) = 1.48 (*p* = 0.47)	**χ^2^ (2) = 9.44 (*p* = 0.01)**
	First	29.8^1^	36.5^1^	23.5^1^
	Second	32.3^1,2^	39.2^1^	25.3^1^
	Third	38.2^2^	42.1^1^	34.6^2^
Parent composition		χ^2^ **(1) = 30.05 (*p* = 0.000)**	**χ^2^ (1) = 24.66 (*p* = 0.000)**	**χ^2^ (1) = 8.83 (*p* = 0.003)**
	Two-parent house	27.8^1^	32.4^1^	23.0^1^
	Other	38.7^2^	46.9^2^	30.9^2^
Dating behaviors				
Held hands		**χ^2^ (1) = 110.54 (*p* = 0.000)**	**χ^2^ (1) = 70.86 (*p* = 0.000)**	**χ^2^ (1) = 21.05 (*p* = 0.000)**
	Yes	53.9^1^	59.3^1^	42.8^1^
	No	27.8^2^	31.7^2^	24.6^2^
Alone with boy-friend/girl-friend		**χ^2^ (1) = 79.97 (*p* = 0.000)**	**χ^2^ (1) = 60.08 (*p* = 0.000)**	**χ^2^ (1) = 6.59 (*p* = 0.01)**
	Yes	55.9^1^	60.6^1^	38.6^1^
	No	29.7^2^	34.7^2^	26.0^2^
Kissed on mouth		**χ^2^ (1) = 74.88 (*p* = 0.000)**	**χ^2^ (1) = 68.16 (*p* = 0.000)**	**χ^2^ (1) = 5.07 (*p* = 0.02)**
	Yes	57.4^1^	69.0^1^	37.0^1^
	No	30.1^2^	34.4^2^	26.1^2^
Said “I love you”		**χ^2^ (1) = 105.95 (*p* = 0.000)**	**χ^2^ (1) = 72.57 (*p* = 0.000)**	**χ^2^ (1) = 17.56 (*p* = 0.000)**
	Yes	55.4^1^	61.7^1^	42.5^1^
	No	28.4^2^	32.4^2^	25.0^2^
Hands under clothes		**χ^2^ (1) = 15.56 (*p* = 0.000)**	**χ^2^ (1) = 9.58 (*p* = 0.002)**	**χ^2^ (1) = 5.36 (*p* = 0.02)**
	Yes	64.7^1^	73.7^1^	53.3^1^
	No	32.6^2^	38.7^2^	26.6^2^
Peer norms of sex initiation^b^		χ^2^ **(1) = 269.02 (*p* = 0.000)**	**χ^2^ (1) = 188.72 (*p* = 0.000)**	**χ^2^ (1) = 75.39 (*p* = 0.000)**
	None	24.0^1^	27.7^1^	21.0^1^
	1 or more	62.3^2^	72.9^2^	21.0^2^

Prevalence reported for predictor variables represent overall (combined oral, vaginal, and anal) sex initiators as weighted percentages. Bold chi-square values represent significant group differences. Different numerical superscripts across column cells for that predictor represent significant differences across response options for the given predictor of interest (bolded in first column).

^a^Sex initiation as reported by 10th grade.

^b^Peer norms of sex initiation indicate the percentage who reported at least one friend to have initiated vaginal intercourse at 7th grade.

### Sexual intercourse initiation differences

Group differences in sexual intercourse initiation are detailed in Table 2. Highlighting significant differences in sexual intercourse initiation across some of the variables of interest here, 1st- [OR = 0.59, 95% CI (0.38, 0.90), *p* = 0.02] and 2nd-generation [OR = 0.64, 95% CI (0.46, 0.90), *p* = 0.01] girls were about half as likely to have initiated sexual intercourse by 10th grade in comparison to 3rd-generation girls. There were no significant differences for Latino boys across the generational status. Girls and boys who reported holding hands at 5th grade [girls OR = 1.77, 95% CI (1.10, 2.85), *p* < 0.001; boys OR = 1.56, 95% CI (1.00, 2.47), *p* = 0.05], as well as those who perceived at least one friend to have had sexual intercourse at 7th grade [girls OR = 3.66, 95% CI (2.47, 5.43), *p* < 0.001; boys OR = 7.17, 95% CI (5.12, 10.05), *p* < 0.001], were more likely to have initiated sexual intercourse by 10th grade. Also, boys who engaged in kissing [OR = 2.07, 95% CI (1.32, 3.24), *p* < 0.01] and saying “I love you” [OR = 1.65, 95% CI (1.01, 2.69), *p* < 0.05] were more likely to have initiated sexual intercourse by 10th grade.

Additionally, boys who reported communicating with their mothers [OR = 0.79, 95% CI (0.65, 0.97), *p* = 0.02] and with their fathers [OR = 0.75, 95% CI (0.61, 0.94), *p* = 0.01] in 5th grade about waiting to have sex were significantly less likely to have initiated sexual intercourse by 10th grade. Girls who reported communicating about what sex is with their mothers in 5th grade were significantly less likely to have initiated sexual intercourse by 10th grade [OR = 0.71, 95% CI (0.51, 0.98), *p* = 0.04]. Girls and boys who reported higher positive attitudes toward sex with a casual friend in 7th grade [girls OR = 1.49, 95% CI (1.16, 1.92), *p* < 0.01; boys OR = 1.29, 95% CI (1.03, 1.61), *p* = 0.03] and acceptability of sex among people who are over 18 and not married [girls OR = 1.25, 95% CI (1.00, 1.57), *p* < 0.05; boys OR = 1.35, 95% CI (1.12, 1.63), *p* < 0.01] were more likely to have initiated sexual intercourse by 10th grade. Also, boys who reported in 7th grade more positive attitudes about sex with a condom [OR = 1.54, 95% CI (1.28, 1.85), *p* < 0.001] were more likely to have initiated sexual intercourse by 10th grade.

### Multi-group comparisons of the structural model across generational status

**FIGURE 2:**
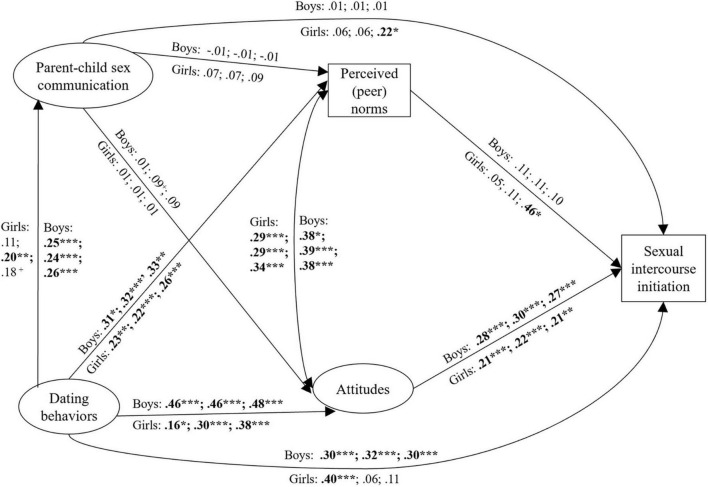
Standardized path coefficients (standard errors) arc presented for the partially constrained structural model across generational status groups (standardized results). Path coefficients for 1st-, 2nd-, and 3rd- generation status groups are presented in that order. Path coefficients above or to the right of paths represent results for Latino boys. Path coefficients below or to the left of paths represent results for Latina girls. Age at grade, household income, and parental household composition were controlled for on the exogenous and outcome variables. Factor loadings and residuals of the measurement model are provided in Table 3. Bold indicates significant coefficients. *p < 0.05, **p < 0.01, ***p < 0.001.

As detailed in Supplementary Appendix A, the measurement model testing using CFA confirmed that the observed variables could be used as expected for the indicated latent variables. Using this measurement model then, first a baseline structural model was tested separately for each generational status group among girls and boys, each of which showed adequate fit among both girls and boys across 1st-, 2nd, and 3rd-generation groups. All fit statistics are reported in Table 3. Based on the retained invariant measurement models (see Supplementary Appendix A for details), we tested the invariance of path coefficients, separately among girls and boys. First, joint unconstrained models (i.e., path coefficients are allowed to vary freely across groups) for all generational status groups were estimated. As detailed in Table 3, the structural model (M1) that freely estimated all paths across generational status groups showed an acceptable fit for both girls and boys. Second, a partially constrained model (M2) in which only the paths that were either significant or non-significant uniformly for all generational status groups in M1 were constrained to be equal across generational status groups. All other paths, including covariances among control variables, were left freely estimated (Kline, 2005). Among girls, the partially constrained model (M2) had a good fit to the data, as shown in Table 3, and the χ^2^ difference test comparing the partially constrained model (M2) to the unconstrained model (M1) indicated they were not significantly different: Δχ^2^ (*df*) = 34.05 (10), *p* < 0.001. Among boys, the partially constrained model (M2) also had a good fit to the data, as shown in Table 3, and the χ^2^ difference test comparing the partially constrained model (M2) to the unconstrained model (M1) indicated they were significantly different: Δχ^2^ (*df*) = 49.38 (18), *p* < 0.001. Thus, the partially constrained model (M2) was selected as the final model for both girls and boys because it was more parsimonious. The resulting path coefficients for this model are shown in Figure 2.

**TABLE 3:** Structural equation modeling fit statistics for baseline and multi-group comparison models.

	Latina girls				Latina boys			
	*χ^2^*	CFI	TLI	RMSEA	*χ^2^*	CFI	TLI	RMSEA
**Baseline model separately by groups**								
First generation	262.17	0.95	0.94	0.04	280.22	0.95	0.94	0.05
Second generation	362.23	0.97	0.97	0.03	297.46	0.98	0.97	0.03
Third generation	299.65	0.95	0.94	0.04	247.95	0.97	0.96	0.04
**Multi-group comparison**								
Model 1 (M1): Joint unconstrained models (i.e., path coefficients are allowed to vary freely across groups) for all generational status groups	931.48	0.97	0.96	0.04	894.54	0.97	0.97	0.04
Model 2 (M2): A partially constrained model, in which only the paths that were either significant or non-significant uniformly for all generational status groups in M1 were constrained to be equal across generational status groups. All other paths were left freely estimated	914.99	0.97	0.97	0.03	845.16	0.98	0.97	0.03

### Generational status differences in specific paths of the model

As a final test of significant differences in path coefficients among the generational status groups separately for girls and boys, several additional models were tested in which paths that differed in significance across generational status groups were constrained to be equal across groups (Kline, 2005). Each path was tested individually based on the partially constrained scalar invariant model (M2; Kline, 2005). Among Latino boys, this strategy revealed that paths from dating behaviors to intentions [Δχ^2^ (*df*) = 69.07 (1), *p* < 0.001], attitudes to intentions [Δχ^2^ (*df*) = 21.06 (1), *p* < 0.001] and sexual intercourse initiation [Δχ^2^ (*df*) = 20.53 (1), *p* < 0.001], and intentions to sexual intercourse initiation [Δχ^2^ (*df*) = 24.63 (1), *p* < 0.001] differed significantly between 3rd- and 1st-generation groups, resulting in a significant χ^2^ differences when compared to the partially constrained model (M2). With the exclusion of the path from dating behaviors to intentions, which also differed significantly between 2nd- and 3rd-generation groups [Δχ^2^ (*df*) = 19.25 (1), *p* < 0.001], these paths also differed significantly between 1st- and 2nd-generation groups [attitude to intentions, Δχ^2^ (*df*) = 10.52 (1), *p* < 0.01; attitude to sexual intercourse initiation, Δχ^2^ (*df*) = 4.55 (1), *p* < 0.05; and intentions to sexual intercourse initiation, Δχ^2^ (*df*) = 32.23 (1), *p* < 0.001]. Among Latina girls, the path from peer norms to sexual intercourse initiation [Δχ^2^ (*df*) = 6.09 (1), *p* < 0.05] differed significantly between 1st- and 3rd-generation groups, resulting in significant χ^2^ difference when compared to the partially constrained model (M2). Additionally, the path from dating behaviors to intentions [Δχ^2^ (*df*) = 11.25 (1), *p* < 0.001] differed significantly between 1st- and 2nd-generation Latina girls.

The final model (see Figure 2) indicated that for all generational status groups of boys as well as 2nd- and 3rd-generation girls, dating behaviors were associated with parent-child sex communication in 5th grade. Among boys of all generational statuses and 1st-generation status girls, dating behaviors in 5th grade predicted 10th-grade sexual intercourse initiation. Significant direct path coefficients are shown in Figure 2. Additionally, mediational paths were tested. For all generational status groups, attitudes mediated the association between dating behaviors and sexual intercourse initiation for girls (1st-generation β = 0.03, S.E. = 0.02, *p* < 0.05; 2nd-generation β = 0.07, S.E. = 0.02, *p* < 0.01; 3rd-generation β = 0.08, S.E. = 0.03, *p* < 0.01) and boys (1st-generation β = 0.13, S.E. = 0.03, *p* < 0.01; 2nd-generation β = 0.14, S.E. = 0.03, *p* < 0.01; 3rd-generation β = 0.13, S.E. = 0.03, *p* < 0.01). Moreover, among 3rd-generation girls, peer norms mediated the association between dating behaviors and sexual intercourse initiation (β = 0.12, S.E. = 0.07, *p* < 0.05). For 1st-generation Latino boys only, attitudes and intentions mediated the association between dating behaviors and sexual intercourse initiation. Specifically, attitudes mediated the association between dating behaviors and intentions (β = 0.20, S.E. = 0.08, *p* = 0.01), intentions mediated the association between attitudes and sexual intercourse initiation (β = 0.19, S.E. = 0.08, *p* < 0.05), and finally, attitudes and intentions together mediated the association between dating behaviors and sexual intercourse initiation (β = 0.08, S.E. = 0.04, *p* < 0.05). Finally, among 2nd-generation boys, attitudes mediated the association between parent-child sex communication and sexual intercourse initiation (β = 0.03, S.E. = 0.02, *p* < 0.05), which, in turn, mediated the association between dating behaviors and sexual intercourse initiation (β = 0.01, S.E. = 0.01, *p* < 0.05).

## Discussion

This study aimed to compare generational status and gender differences in the longitudinal predictions from parent-child sex communication and dating behaviors to sex initiation (oral, vaginal, and anal) 5 years later, and how these associations are mediated by perceived peer norms, intentions, and attitudes regarding sex among Latina/o adolescent girls and boys. This is the first study to concurrently examine the longitudinal influence of parent-child discussions about sex and dating behaviors on subsequent sex initiation as mediated by various decision-making factors among Latina/o adolescents in the US with different family migration histories. As the largest ethnic minority group in the US, Latina/o youth are particularly vulnerable in that Latina/o adolescents tend to initiate sex earlier than non-Hispanic White adolescents in the US, and are less likely to use condoms consistently leading to an increased risk of contracting STIs and higher rates of unintentional pregnancy (Morales-Aleman and Scarinci, 2016). The findings from this study clarify how differences in early sex initiation and related attitudes, intentions, and subjective peer norms are shaped in early adolescence by dating behaviors and parental communication about sex to identify whether each process increases or reduces the risk of early sex initiation. As a multisite study, longitudinal study which captures changes in developmental hallmarks related to sexual behaviors, data from the Healthy Passages™ allow us to highlight the variations across generational status among Latina/os and throughout early- to mid-adolescence. In comparison, most other studies often neglect acculturation processes (Titzmann and Lee, 2018) related to generational status differences and are limited to observing behaviors in later adolescence.

Our first hypothesis that less acculturated Latina/os would report a lower prevalence of sexual intercourse initiation was supported for girls, in that less acculturated 1st- and 2nd-generation Latinas reported a lower prevalence of sexual intercourse initiation than 3rd-generation girls. However, this was not supported by Latino boys. These generational status differences among girls and the lack of evidence for the immigrant paradox among boys are consistent with previous findings (Guarini et al., 2011; Haderxhanaj et al., 2015). Also consistent with previous research, our results demonstrate that Latino boys have higher levels of sexual initiation than Latina girls (Guarini et al., 2011).

Our findings partially supported hypotheses 2 and 3 about the roles of early parent-child sex communication and dating behaviors on sexual intercourse initiation 5 years later, and the mediation of decision-making processes in these relationships among Latina/o adolescents. Specifically, the overall model, which had a good fit for all groups, demonstrated that for girls of 1st generation and boys of all generational status groups, engaging in dating behaviors in 5th grade was associated with a greater likelihood of sexual intercourse initiation by 10th grade. Moreover, for girls and boys of all generational status groups, engaging in pre-adolescent dating behaviors was associated with a greater likelihood of perceiving peers having initiated sexual intercourse and higher positive attitudes about sexual intercourse. The association between dating and sex initiation is in line with previous work which focused on comparing Black and non-Black adolescent groups, which found similar associations between early dating relationships and sex initiation among males and females in the US (Cooksey et al., 2002). As no recent studies have replicated previous research to examine the association between general dating patterns and sex initiation, this study expands our understanding of the link between early dating patterns and sex initiation by focusing on Latina/o adolescents and identifying decision-making processes, specifically attitudes, that mediate this relationship. Also, for all groups, such positive attitudes were associated with a greater likelihood of sexual intercourse initiation. We discuss these findings in turn.

First, we found that early dating behaviors were positively associated with subsequent sexual intercourse initiation for most groups. It is important to note that early dating may be driven by a third variable not explored in this study, for example, age of pubertal onset which is often associated with various health behaviors and outcomes (Brooks-Gunn, 2021). Yet, it may be that the earlier an adolescent begins to engage in dating behaviors, the longer the period of opportunity is for exploring sexual behaviors (Furman et al., 1999). Early romantic, or even exploratory, relationships may build confidence in sexual interactions (Wood et al., 2008) and, further, reinforce interest in sexual behaviors (Birnbaum and Reis, 2019). Moreover, the direct association between dating behaviors and sexual intercourse initiation was consistent across generational status for boys, but was only significant for 1st-generation girls. It may be that, for boys, there are no differences in expectations whether primarily adhering to traditional Latino or US cultural norms regarding dating and sex. Conversely, for girls, the protective immigrant experience appears to erode with early dating experience, as other protective factors erode with acculturation (Finch, 2019). Early dating behaviors among less acculturated girls may increase their engagement in sexual interactions (Raffaelli and Ontai, 2001). Essentially, by breaking with traditional cultural values regarding dating, Latina girls may not experience the protective mechanism typically associated with lesser acculturation, similar to the association between bicultural stress and problem behaviors (e.g., aggression and substance use; Bosma et al., 2019). Perhaps, differences in how less acculturated girls are brought up regarding dating and sex in comparison to more acculturated girls may not be as effective in delaying sexual intercourse initiation (Haglund et al., 2019).

Second, whereas the association between dating behaviors and sexual intercourse initiation was mediated by attitudes and intentions for 1st-generation Latino boys, it was only mediated by attitudes for 2nd- and 3rd-generation Latina girls and Latino boys. This association was also mediated by peer norms for 3rd-generation Latina girls. Also, this association was mediated by both parent-child sex communication and attitudes for 2nd-generation boys. For all groups of boys and girls, attitudes were both a direct predictor and mediator of sexual intercourse initiation. This suggests that independent of acculturation differences, sexual attitudes are an important factor in the sexual decision-making process. Moreover, Latina/os typically have more restrictive attitudes about sex than non-Latina/os (Eisenman and Dantzker, 2006), which appear to differ across generational status depending on the specific sexual attitude in our study (see Table 1). Attitudes for Latina/o youth who are more exposed to US dominant cultural norms may be influenced more by the dominant culture than is the case for those with less exposure (Scull and Malik, 2019; Blanc, 2021), who may be more aligned to attitudes of their cultural heritage.

Our results also demonstrated differences across Latina girls’ generational status groups regarding peer norms and intentions. These generational status differences in mediating decision-making factors may suggest that girls of different generational statuses may rely on different decision-making processes related to sexual intercourse, depending on their acculturation. In fact, cognitions may shift in acculturation processes and account for some of the observed generational status differences (Abraído-Lanza et al., 2016). For example, more acculturated adolescents tend to have greater intentions to engage in risky behaviors generally than those less acculturated (Blake et al., 2001). Perhaps, less acculturated Latino/a youth are more likely to hold sexual decision-making values that are more aligned with traditional cultural norms than more acculturated youth, which may be a protective mechanism. Yet, our findings would also suggest that, despite aligning their attitudes, intentions, and norms to traditional cultural scripts, the protective mechanism often associated with holding on to traditional cultural views may be eroded when adolescents engage in dating behaviors early in life. This may be because engaging in such behaviors, in a sense, releases adolescents from traditional cultural expectations against early romantic exploration (Raffaelli and Ontai, 2001).

Also, for more acculturated 3rd-generation Latina girls, perceiving their peers to have initiated sexual intercourse was associated with their own sexual intercourse initiation. This finding is in line with previous work that suggests peers play a stronger role in sexual intentions for US-born Latina/o youth (Killoren et al., 2011b). Less acculturated Latinas may be more resistant to peer influences, such as peer pressure, than those more acculturated. Thus, erosion of the protection associated with holding on to cultural traditions among Latina/o youth appears to be reflected in decision-making processes (i.e., peer norms and attitudes).

Finally, generational status differences may suggest that how adolescents decide to have sexual intercourse differs according to their cultural experiences via acculturation processes. Differences in decision-making strategies may be the result of greater adherence to traditional cultural views of sexual behavior among some groups, which, in turn, is a strong predictor of behaviors (Villar and Concha, 2012). Also, the consistent emphasis on attitudes regarding sexual intercourse initiation as a mediator in this association among most generational status groups may suggest that cultural conflicts often experienced among many Latino/a youth are most reflected in attitudes (e.g., Villarruel et al., 2004). That is, as Latino/a youth navigate the often-opposing views of traditional cultural views and norms and those of the dominant US culture regarding sexual and dating norms and behaviors (e.g., Roysircar-Sodowsky and Maestas, 2000), their attitudes toward dating and sex may be most influenced by these experiences. Thus, our model results suggest a nuanced process of sexual decision-making is influenced by early dating behaviors and acculturative processes.

### Limitations

Foremost, the observational design did not allow us to establish causal links between variables. Also, we could not account for changes across time in decision-making processes because observed variables in 7th grade were not measured in 5th grade. Generational status is a single indicator of acculturation, which is a complex process. Moreover, this was measured without consideration for the migration status of a second parent, or time in the US for either child or parent, which can have differential effects (Coll and Marks, 2012). Being restricted to single-item measurements of constructs, such as intentions and peer norms, is an additional limitation. Because Latina/o participants were sampled from only two geographic regions, findings might not generalize to other regions in the US. Further, because our Latino sample has familial roots primarily in Mexico and Central America, and because of its within-group heterogeneity, caution must be exercised in generalizing to Latino groups with other origins. Age of sexual intercourse initiation was not measured with the traditional cut-off score of early sex initiation (Zimmer-Gembeck and Helfand, 2008). Measures of intentions, perceived peer norms, and attitudes relied on references to vaginal intercourse only; thus, we were unable to address the comprehensive influence of these sexual decision-making processes on oral and anal sex.

### Future research

Despite adolescents’ diverse sexual experiences, including oral and anal intercourse, most studies have focused only on vaginal intercourse. We attempted to bridge this gap by examining sexual initiation across oral, vaginal, and anal sex. However, we were unable to examine differences across sexual orientations. Future research should endeavor to do so, as well as examine other sexual risk behaviors (e.g., contraceptive use) and outcomes (e.g., the prevalence of STIs).

Our findings support the link between parental communication about sex with pre-adolescent dating behaviors and, subsequently, sex initiation. Future research should explore communication specifically about dating behaviors, including how and when parents discuss dating behaviors with their children, as well as the longitudinal association of these factors with subsequent adolescent sexual behaviors. Moreover, discussions about sex may differ depending on the gender composition of parent-child dyads (Evans et al., 2020; Pasqualini and De Rose, 2020). In fact, our descriptive results demonstrate that mothers discussed sex topics at a higher prevalence than fathers. Future studies should explore how opposite-gender parent-child dyads address sex discussions among Latino families. Expanding our understanding of parent-child communication in relation to early dating behaviors among US Latina/o adolescents across various measures of sexual behaviors may help further clarify why some Latina/o adolescents are at higher risk.

### Implications

Once replicated, these findings may guide family-level prevention strategies aimed at delaying sexual intercourse initiation among Latina/o youth. Specifically, Latino parents need guidance to discuss dating and sexual behaviors with their pre-adolescent children (Guilamo-Ramos and Bouris, 2008; Wilson et al., 2010; Pariera and Brody, 2018), as dating behaviors should be addressed early in Latino families. These behaviors should be addressed prior to 5th grade, which appears to be a critical period of dating initiation for Latina/o youth. This may be particularly important among less acculturated Latina girls. Sexual risk prevention programs may be adapted to address dating behaviors. Yet, targeted prevention strategies must balance between respecting Latino parents’ traditional values while also providing sufficiently informative guidance to their children for navigating sexual situations they may encounter in their dating relationships. Also, programs that focus on attitudes may be best at reaching a larger group of Latina/o youth and most effective at delaying sex initiation (Morrison et al., 2018). Multifaceted strategies that aim to address each decision-making process may also be successful for Latina/o youth from diverse acculturative experiences (Lee et al., 2018).

## Data availability statement

The data analyzed in this study is subject to the following licenses/restrictions: The data analyzed in this study is not publicly available due to concerns of confidentiality for participants under the age of 18 years but are available upon request from the authors. Requests to access these datasets should be directed to JW, jwallander@ucmerced.edu.

## Ethics statement

The studies involving human participants were reviewed and approved by the institutional review boards at each study site (University of California Los Angeles; University of Texas at Austin; University of Alabama at Birmingham) and the Centers for Disease Control and Prevention approved the study. Written informed consent to participate in this study was provided by the participants’ legal guardian/next of kin.

## Author contributions

PC was the lead author in conceptualizing the idea, hypotheses for the manuscript as well as running the analyses and writing the manuscript. JW, ME, and MS were the original contributors of the Healthy Passages project and provided additional editing and writing for the manuscript. All authors contributed to the article and approved the submitted version.
