# Supplementary material for: Longitudinal associations of parent-child communication, dating behaviors, decision-making processes, and sex initiation among United States Latina/o adolescents
Source: Front Psychol. 2022 Aug 12;13:897311. doi: 10.3389/fpsyg.2022.897311 (PMC9413066; doi:10.3389/fpsyg.2022.897311)
Supplement: Supplementary file 1 [file Data_Sheet_1.doc]

**Appendix A**

**Evaluation of the Measurement Model**

Confirmatory factor analysis (CFA) of latent variables indicated adequate fit to the data for: dating behaviors for both girls (χ^2^ = 14.91, *df* = 5, CFI = .99, TLI = .99, RMSEA = .05) and boys (χ^2^ = 6.31, *df* = 5, CFI = .99, TLI = .99, RMSEA = .02); parent-child sex communication for both girls (χ^2^ = 165.12, *df* = 9, CFI = .96, TLI = .94, RMSEA = .04) and boys (χ^2^ = 342.24, *df* = 9, CFI = .92, TLI = .95, RMSEA = .05); and attitudes about sex for both girls (χ^2^ = 10.77, *df* = 5, CFI = .99, TLI = .99, RMSEA = .09) and boys (χ^2^ = 12.04, *df* = 5, CFI = .99, TLI = .99, RMSEA = .04). Factor loadings for the measurement model are shown in Appendix B.

**Invariance Testing Procedure**

After each latent variable was supported in the confirmatory factor analysis, invariance (or equivalency) tests of the measurement model were conducted across generational status groups. This step investigates whether the factor structure of the measurement model for latent variables was equivalent across groups. Thus, invariance testing was conducted comparing consecutively constrained models that build on each successful level of previous invariance test, reflecting (1) configural, (2) metric, and (3) scalar invariance (Byrne, 2012). Configural invariance indicates whether observed variables conform to the same feature across groups, metric invariance determines whether loadings for each observed variable on its posited latent factor are equivalent, and finally, scalar invariance examines whether the intercepts of each observed variable are equal. In comparing models, the Chi square difference test was used.

**Invariance of the Measurement Model across Generational Status**

Invariance of the measurement model across generational status groups was examined by comparing consecutively constrained invariant models: Among girls, initial invariance test results showed that the comparison between the unconstrained configural invariance model (Model 1; χ^2^ = 916.98, *df* = 667) and metric invariance model (Model 2; factor loadings and variances constrained) yielded a significant difference (Δχ^2^ (*df*) = 29.72 (6), *p* < 0.01). Then, one parameter was identified as non-invariant (i.e., parent-child sex communication variance) and released in Model 2a. Subsequently, the comparison between Model 2a and the scalar invariance model (Model 3; thresholds constrained) did not yield a difference (Δχ^2^ (*df*) = 0.0 (0), *ns*).

Among boys, results showed that the comparison between the unconstrained configural invariance model (Model 1; χ^2^ = 895.88, *df* =602) and metric invariance model (Model 2; factor loadings and variances constrained) did not yield a significant difference: (Δχ^2^ (*df*) = 1.34 (6), *p* >.05). Also, the comparison between Model 2 and the scalar invariance model (Model 3; thresholds constrained) did not yield a significant difference: (Δχ^2^ (*df*) = 0.0 (0), *ns*). Thus, a partial scalar invariance (equivalence) for the measurement model across generational status groups for girls and a full scalar invariance measurement model across generational status for boys was obtained. and was used in multi-group comparisons (Byrne, Shavelson, & Muthén, 1989).
