# Supplementary material for: Longitudinal associations of parent-child communication, dating behaviors, decision-making processes, and sex initiation among United States Latina/o adolescents
Source: Front Psychol. 2022 Aug 12;13:897311. doi: 10.3389/fpsyg.2022.897311 (PMC9413066; doi:10.3389/fpsyg.2022.897311)
Supplement: Supplementary file 2 [file Table_1.doc]

**Appendix B**

Table B1. Correlations, means, standard deviations of observed variables, and standardized factor loadings and residuals of the measurement model.

|  | **1** | **2** | **3** | **4** | **5** | **6** | **7** | **8** | **9** | **10** | **11** | **12** | **13** | **14** | **15** | **16** | **17** | **18** | **19** | **20** | **21** | **22** | **23** |
| --- | --- | --- | --- | --- | --- | --- | --- | --- | --- | --- | --- | --- | --- | --- | --- | --- | --- | --- | --- | --- | --- | --- | --- |
| **1** | 1 | .29 | -.03 | -.01 | -.14 | -.03 | -.01 | -.05 | -.08 | -.07 | -.08 | .01 | .05 | .04 | .04 | .05 | .02 | -.04 | -.06 | -.01 | -.01 | .02 | .04 |
| **2** | .31 | 1 | -.05 | -.05 | .20 | .01 | .01 | -.10 | -.03 | .01 | -.12 | -.04 | -.03 | -.08 | -.02 | -.03 | -.03 | -.05 | -.06 | -.07 | -.05 | -.04 | -.05 |
| **3** | -.07 | -.11 | 1 | .83 | -.16 | -.05 | -.02 | -.03 | -.01 | -.01 | -.01 | .10 | .05 | .13 | .12 | -.02 | .19 | .20 | .14 | .25 | .22 | .14 | .11 |
| **4** | -.05 | -.08 | .79 | 1 | -.14 | -.01 | -.02 | -.01 | .01 | .02 | -.01 | .11 | .05 | .14 | .13 | .02 | .27 | .19 | .15 | .24 | .21 | .14 | .23 |
| **5** | -.13 | .06 | -.13 | -.10 | 1 | .05 | .03 | .02 | .03 | .10 | .02 | -.12 | -.11 | -.10 | -.12 | -.01 | -.10 | -.15 | -.11 | -14 | -.15 | -.09 | -.15 |
| **6** | -.06 | -.09 | .01 | -.02 | .04 | 1 | .56 | .36 | .56 | .44 | .28 | .06 | .01 | .04 | .02 | .03 | .01 | .12 | .11 | .14 | .13 | .12 | .01 |
| **7** | -.05 | -.06 | .07 | .01 | -.02 | .62 | 1 | .46 | .51 | .61 | .40 | .14 | .10 | .05 | .08 | .01 | -.01 | .14 | .02 | .11 | .09 | .12 | .06 |
| **8** | -.08 | -.09 | .08 | .04 | -.04 | .45 | .55 | 1 | .37 | .39 | .70 | .12 | .05 | .09 | .05 | -.05 | .08 | .06 | .00 | .06 | .02 | .02 | .10 |
| **9** | -.10 | -.07 | .02 | .02 | .06 | .46 | .45 | .32 | 1 | .62 | .41 | .14 | .10 | .08 | .10 | .02 | .06 | .14 | .11 | .14 | .14 | .12 | .05 |
| **10** | -.08 | -.08 | -.01 | -.01 | .03 | .38 | .52 | .36 | .64 | 1 | .49 | .15 | .12 | .13 | .09 | .03 | .04 | .11 | .06 | .07 | .10 | .09 | .05 |
| **11** | -.11 | -.06 | .03 | -.02 | .09 | .35 | .42 | .57 | .47 | .55 | 1 | .14 | .06 | .09 | .09 | .01 | .10 | .07 | .05 | .11 | .05 | .04 | .11 |
| **12** | .05 | .01 | .20 | .19 | -.09 | .08 | .13 | .08 | .01 | .05 | .07 | 1 | .54 | .46 | .59 | .11 | .25 | .27 | .23 | .27 | .27 | .22 | .25 |
| **13** | .09 | .02 | .13 | .17 | -.07 | .03 | .03 | -.01 | -.02 | .03 | -.02 | .56 | 1 | .45 | .51 | .15 | .21 | .26 | .20 | .20 | .24 | .19 | .23 |
| **14** | .02 | .02 | .15 | .13 | -.04 | .04 | .09 | .08 | .03 | .02 | .05 | .39 | .47 | 1 | .45 | .22 | .29 | .28 | .22 | .25 | .28 | .20 | .25 |
| **15** | .04 | -.02 | .15 | .13 | -.04 | .08 | .09 | .05 | .03 | .03 | .04 | .62 | .45 | .40 | 1 | .15 | .23 | .25 | .20 | .23 | .26 | .18 | .25 |
| **16** | .01 | .02 | -.01 | .02 | -.01 | .05 | .03 | .02 | -.03 | .03 | .02 | .20 | .25 | .19 | .21 | 1 | .10 | .12 | .11 | .13 | .10 | .07 | .09 |
| **17** | .07 | .01 | .20 | .26 | -.11 | .10 | .09 | .09 | .11 | .01 | .09 | .24 | .15 | .18 | .22 | .07 | 1 | .45 | .39 | .43 | .46 | .37 | .41 |
| **18** | .03 | -.02 | .17 | .17 | -.08 | .04 | .02 | .05 | .07 | .02 | .03 | .18 | .18 | .09 | .12 | .02 | .32 | 1 | .59 | .62 | .66 | .52 | .34 |
| **19** | .05 | -.02 | .17 | .17 | .01 | .04 | .05 | .04 | .07 | .05 | .04 | .15 | .12 | .10 | .12 | .01 | .26 | .53 | 1 | .65 | .68 | .49 | .30 |
| **20** | -.03 | -.04 | .23 | .23 | -.06 | .02 | .02 | .04 | .05 | .01 | .01 | .21 | .13 | .07 | .17 | .04 | .38 | .54 | .58 | 1 | .75 | .52 | .36 |
| **21** | -.04 | -.07 | .21 | .22 | -.05 | .04 | .03 | .03 | .09 | .02 | .02 | .22 | .17 | .09 | .20 | .05 | .37 | .53 | .56 | .74 | 1 | .57 | .36 |
| **22** | .03 | .05 | .17 | .14 | -.10 | .03 | .04 | -.01 | .02 | -.01 | .03 | .19 | .13 | .13 | .17 | .07 | .32 | .38 | .36 | .48 | .46 | 1 | .35 |
| **23** | -.06 | .03 | -.03 | -.02 | .01 | .05 | .15 | -.02 | .06 | .02 | -.04 | .01 | .02 | .01 | .01 | -.02 | .04 | .12 | .04 | .12 | .07 | .16 | .16 |
| **24** | .08 | .00 | .16 | .24 | -.09 | .04 | .10 | .07 | .06 | .05 | .03 | .14 | .08 | .07 | .13 | .07 | .26 | .24 | .17 | .23 | .21 | .20 | 1 |
| **^+^Mean** | 2.03 | 2.04 | 10.65 | 16.08 | 0.52 | 1.58 | 1.42 | 1.77 | 1.46 | 1.36 | 1.66 | 0.27 | 0.19 | 0.14 | 0.24 | 0.02 | 0.26 | 2.09 | 2.10 | 2.33 | 2.20 | 2.60 | 0.39 |
| **SD** | .63 | 1.65 | .68 | .65 | .50 | .67 | .64 | .85 | .66 | .62 | .81 | .45 | .39 | .35 | .42 | .13 | .44 | .99 | .99 | 1.03 | 1.01 | .94 | .49 |
| **^+^LDG** | — | —^a^ | —^a^ | —^a^ | —^a^ | [.73 | .81 | .86 | .81 | .86 | .84]^b^ | [.98 | .85 | .84 | .85 | .85]^c^ | —^e^ | [.81 | .83 | .87 | .91 | .71]^d^ | —^e^ |
| **S.E.** | — | — | — | — | — | .02 | .02 | .02 | .02 | .02 | .01 | .03 | .02 | .03 | .02 | .02 | —^e^ | .01 | .01 | .01 | .01 | .02 | —^e^ |
| **^++^Mean** | 2.02 | 1.93 | 10.60 | 16.03 | 0.50 | 1.91 | 1.67 | 2.02 | 1.35 | 1.30 | 1.62 | 0.13 | 0.08 | 0.08 | 0.11 | 0.01 | 0.21 | 1.52 | 1.67 | 1.88 | 1.75 | 2.26 | 0.27 |
| **SD** | .64 | 1.61 | .64 | .58 | .50 | .74 | .76 | .89 | .60 | .58 | .82 | .33 | .27 | .27 | .32 | .12 | .41 | .73 | .86 | .98 | .88 | .93 | .44 |
| **^++^LDG** | — | —^a^ | —^a^ | —^a^ | —^a^ | [.76 | .86 | .82 | .82 | .90 | .81]^b^ | [.96 | .89 | .78 | .91 | .63]^c^ | —^e^ | .76 | .79 | .90 | .89 | .63 | —^e^ |
| **S.E.** | — | — | — | — | — | .02 | .02 | .02 | .02 | .03 | .02 | .03 | .03 | .04 | .03 | .06 | —^e^ | .02 | .02 | .01 | .01 | .02 | —^e^ |

Correlations for girls (*n* = 879) are to the bottom and left of the diagonal. Correlations for boys (*n* = 885) are to the right and above the diagonal. LDG = Loading.

All correlations *r* ≥ .06, *p* < .05; *r* ≥ .08, *p* < .01. ^+^Descriptive statistics and standardized factor loadings for boys; ^++^Descriptive statistics and standardized factor loadings for girls.

1 = generational status; 2 = household income; 3 = Age at 5^th^ grade; 4 = Age at 10^th^ grade; 5 = parent household composition; 6 = Mom – how babies are made; 7 = Mom – what is sex; 8 = Mom – wait to have sex; 9 = Dad – how babies are made; 10 = Dad – what is sex; 11 = Dad – wait to have sex; 12 = held hands; 13 = alone with bf/gf; 14 = kissed on mouth; 15 = said “I love you”; 16 = hands under clothes; 17 = peer norms; 18 = sex with casual friend; 19 = sex with bf/gf; 20 = sex acceptable with condom; 21 = sex acceptable in love; 22 = sex acceptable if older than 18 years old; 23 = sexual intercourse initiation.

^a^Control variables.

^b^Standardized loadings for *parent-child sex communication* latent factor.

^c^Standardized loadings for *dating behaviors* latent factor.

^d^Standardized loadings for *attitudes* latent factor.

^e^Categorical items in structural model which do not provide loadings.
